# Peat cutaway properties define after-use options and capacity for climate regulation

**DOI:** 10.1007/s00267-026-02446-9

**Published:** 2026-04-21

**Authors:** Liisa Maanavilja, Oona Allonen, Lasse Aro, Heikki Sutinen, Tuija Vähäkuopus, Anna M. Laine

**Affiliations:** 1 Geological Survey of Finland (GTK), Espoo, Finland; 2 Natural Resources Institute Finland (Luke), Turku, Finland; 3 School of Forest Sciences, University of Eastern Finland, Joensuu, Finland

**Keywords:** Peat extraction, Peat cutaway, Peatland, Land use, Climate impact, Restoration

## Abstract

Mitigating climate change and halting biodiversity loss require sustainable after-use strategies for peat extraction sites. This study evaluates the climate regulation potential of post-extraction land use using two peat cutaway sites in South Ostrobothnia, Finland, as case examples. The land use portfolio examined includes afforestation (conventional and short-rotation), peatland restoration, creation of open-water wetlands, conventional agriculture, reed canary grass cultivation and paludiculture. First, we mapped all suitable areas for each land use at the two case study sites, based on detailed spatial data on surface elevation, peat thickness and subsoil characteristics. Second, we calculated the climate warming impact of each land use based on published greenhouse gas flux and biomass estimates using REFUGE4, a climate impact model. Third, we assessed the climate impact of land-use scenarios aligned with five different objectives: climate regulation, ecological restoration, food production, timber production, and peat-replacing biomass production. This allowed us to explore how landowners’ preferences or policy choices may influence climate outcomes. The results suggest that various climate-wise land use combinations are feasible after peat extraction, depending on site-specific conditions and landowner and policy preferences. We propose that tailoring land use to site characteristics and using strategic damming and ditching to create a multi-habitat landscape can provide both economic and ecological benefits. However, substantial knowledge gaps remain regarding greenhouse gas fluxes from post-extraction land uses.

## Introduction

The climate crisis calls for land uses that protect carbon stocks and increase carbon uptake (IPBES 2019). Peatlands are a globally significant carbon reservoir: although they cover only about three percent of the Earth’s land surface, they store approximately as much carbon as is currently present in the atmosphere (Gorham 1991; Yu et al. 2010; Nichols and Peteet 2019). Drainage-based peatland use releases this carbon into the atmosphere, with a rate influenced by peat decomposability and soil management (IPCC 2014). Peatland exploitation has been particularly intensive in Europe, where ~10% of the original peatland area has been lost and a further 46% is considered degraded due to land use change (UNEP 2023). In response, recent EU policies – such as the Nature Restoration Law, the Common Agricultural Policy 2023–2027, and the Carbon Removal Certification Framework – have brought peatland management to the forefront of land-use planning and governance in the EU (European Parliament and Council of the European Union 2024a, b; Nordbeck et al. 2025).

Among land uses of peatlands, peat extraction is particularly disruptive. Although its global extent is small, its ecological impact is severe (Joosten et al. 2002; Chapman et al. 2003). Peat extraction removes the surface peat layer and vegetation, leaving behind a dry, dark and loose peat surface, often with low nutrient availability. These conditions hinder plant establishment and make the site vulnerable to wind and water erosion (Lavoie et al. 2003; Waddington et al. 2002). After the peat extraction ceases, cutaway areas are typically transferred to other land uses, such as forestry, agriculture or peatland restoration (Räsänen et al. 2023). While abandonment was common in the past, many countries have in recent decades mandated at least basic site rehabilitation measures to support vegetation re-establishment (Karofeld et al. 2017).

National legislation may require mire creation (Canada), rewetting (Germany), or any kind of vegetation re-establishment (Finland). In Finland, where the legislation allows a wide variety of land uses, private landowners aim for profitable uses: afforestation, agriculture, or more recently also solar power (Laasasenaho et al. 2023). The land‑use portfolio examined in this study includes afforestation (conventional and short‑rotation forestry), peatland restoration, creation of open‑water wetlands, conventional agriculture, cultivation of reed canary grass, and paludiculture.

Afforestation can be profitable with several tree species, including Scots pine (*Pinus sylvestris*) for timber (Aro et al. 2020) and short-rotation biomass production of downy birch (*Betula pubescens*) for biomass (Jylhä et al. 2015). Successful afforestation of cutaway peatlands generally requires fertilisation to compensate for the shortage of phosphorus and potassium in the residual peat (Räsänen et al. 2023).

Restoration of cutaway peatlands to wetlands has taken different forms in North America and Europe. In Canada, the moss layer transfer technique was developed for cutover peatlands with the topmost peat extracted for horticulture, to support the development of bog-type vegetation (González and Rochefort 2014). In Europe, deep energy peat extraction has created areas of low topographical position relative to the surrounding landscape, making them more suitable for rewetting into wetlands with open water surfaces (Money 2004). These wetlands are constructed to support waterbird populations and enhance overall biodiversity. *Sphagnum* moss establishment is also possible in deeply extracted cutaways but requires skilful regulation of the water table level (Oestmann et al. 2022).

Agriculture in peat soils in not encouraged in the European Union, as drainage-based cultivation causes substantial greenhouse gas emissions and is targeted for phase-out in EU climate and agricultural policies (European Parliament and Council of the European Union 2024a). However, perennial grass cultivation is tolerated as a better alternative than the cultivation of cereals and other annual crops (Nordbeck et al. 2025). In recent years, demand has grown for biomass crops as peat alternatives – particularly for growing media and animal bedding – with *Phalaris arundinacea* (reed canary grass) emerging as a promising option (Lång et al. 2022).

Paludiculture, the cultivation of crops on wet or rewetted peatlands, has been proposed as a way to reduce greenhouse gas emissions from peat soils while generating income for people (Joosten 2016). Currently, *Phalaris arundinacea* is cultivated under conventional dry conditions, but it is also well adapted to high water table environments, making it a suitable candidate for paludiculture (Nielsen et al. 2024b). *Typha* (cattail) has also been identified as a promising crop for paludiculture (Lahtinen et al. 2022).

After-use types differ in their greenhouse gas (GHG) emissions (Räsänen et al. 2023; Mander et al. 2024). Therefore, the climate regulation potential of a former peat extraction site can be influenced by appropriate land use choices (Laasasenaho et al. 2023). Drained land uses typically emit CO₂ and often N₂O, while CH₄ emissions are low (Maljanen et al. 2010). Wetland restoration or paludiculture reverses this pattern, with increased CH₄ but reduced CO₂ and N₂O emissions (Bianchi et al. 2021; IPCC 2021). The temporal dynamics of these emissions encompass both ecological succession at the site (Nugent et al. 2019) and the atmospheric behaviour of greenhouse gases, particularly CH_4_, which is a potent but short-lived greenhouse gas (IPCC 2021). The net climate impact depends on the balance of the emissions and the time frame over which the impact is assessed. However, site conditions, such as residual peat thickness, elevation, and the quality of peat and subsoil limit the feasibility of different land use options (Picken 2007). These factors vary both within and between peat extraction sites (Picken 2007; Räsänen et al. 2023).

Previous scientific syntheses assessing the climate impact of the after-use of former peat extraction sites have not considered the typical spatial heterogeneity of their site conditions and have neglected or only partially addressed the temporal dynamics of the post-extraction climate impact (Laasasenaho et al. 2023; Mander et al. 2024). Acknowledging both spatial variability and temporal dynamics is crucial for accurately estimating the climate change mitigation potential of these sites and for identifying the most effective measures to support climate-wise after-use.

The aim of this study is to investigate the potential for climate regulation in the after-use of cutaway peatlands, given the site conditions.

First, we mapped all suitable areas for each land use at the two case study sites in Finland, based on detailed spatial data on surface elevation, peat thickness and subsoil characteristics. Second, we compiled and synthesised published data on greenhouse gas fluxes associated with different after-use types in boreal and cool temperate climates and calculated their climate impact using REFUGE4, a climate impact model. Third, we assessed the climate impact of land-use scenarios aligned with five different objectives: (a) climate regulation, (b) ecological restoration, (c) food production, (d) timber production, and (e) peat-replacing biomass production. This allowed us to explore how landowners’ preferences or policy choices may influence climate outcomes.

## Material and Methods

### Mapping Areas Suitable for Different After-uses at Case Study Sites

We identified the elevation gradient (indicating the potential for water table management), residual peat thickness, and mineral subsoil type as key site conditions that constrain the after-use options, each with specific thresholds relevant to different after-use types (Aro et al. 1997; Picken 2007; Salo and Savolainen 2008; Luhtala 2021; Latva-Krekola and Luhtanen 2021; Aro et al. 2023). To define the thresholds, we drew on both the literature and expert opinions from Finnish peatland scientists affiliated with the Geological Survey of Finland GTK, Natural Resources Institute Finland Luke, Tapio (a state-owned forestry consultancy) and Finnish Environment Institute Syke.

We conducted detailed field surveys on these site properties at two peat extraction sites in South Ostrobothnia, Finland: Kampinneva (N = 6992285, E = 314530, ETRS-TM35FIN), covering 123 ha, and Ohraneva (N = 7012117, E = 314264, ETRS-TM35FIN), covering 170 ha. Peat extraction was still ongoing in some sections at both sites.

A detailed digital elevation model (DSM) of the surface was obtained through photogrammetric orthography using a WingtraOne fixed-wing UAV, flown at an altitude of 120 m, with post-processing kinematic (PPK) positioning, integrated with a Sony RX1RXII 42 MP camera. Post-processing utilized RINEX post-calculation data and WingtraHub and Pix4D software. The resulting DSM had a pixel size of 1.6 cm and accuracy of less than 10 centimeters.

To study peat thickness and the subsoil, the elevations of the surface and the subsoil bottom were surveyed using an SIR-4000 (Geophysical Survey Systems, Inc.) ground penetrating radar (GPR) with a 200 MHz antenna, integrated into an all-terrain vehicle. The survey was conducted continuously from every second strip, resulting in a survey interval of ~40 m. Position measurements were taken every metre using RTK-GPS with a VRS system (Trimble R12i/T10), achieving coordinate accuracy of less than 5 centimeters. Reference peat drill samples were taken to determine accurate interpretation values for the peat velocity and to verify the subsoil type. The GPR data were processed and interpreted using RoadDoctor (Roadscanners) and 3D-Win (Novatron) software.

Mapping suitable after-use types at the sites was based on GIS analysis. First, we mapped the drainage and rewetting potential of the site. We identified (a) the areas constrained to dry land uses as rewetting them was impossible or very difficult without impacting (flooding) land surrounding the cutaway, (b) the areas constrained to wet uses as they were hard to keep dry without pumping, and (c) the areas suitable for wet or dry use as they could be re-wetted or kept dry with drainage ditch management and dam constructions.

This assessment involved identifying 1) the bottom level of the main outflowing drainage ditch, which could be either a water surface or the ditch bottom: areas below or less than 50 cm above that were constrained to wet uses, and 2) the surface level of border embankments relative to the surface level of the surrounding areas: the areas were constrained to dry uses if high water table would lead to water flowing into the surrounding areas. The shallow areas below or less than 50 cm above main drainage ditch were deemed areas that would likely have open water. Aerial image of the areas corresponded with this interpretation. Areas above this, but not higher than border embankments were deemed as suitable for peatland restoration. The actual extent of open water could be larger than estimated here if the water level were raised to the maximum possible level. However, the large open-water extent can be mitigated using embankments and terraces within the restoration area, as none of the scenarios prioritize open-water wetlands (see Section “Climate impact of different land use scenarios at the case study sites”).

This analysis was carried out in ArcGIS Pro by extracting raster cells within a specified elevation range above sea level. For the cutaway area, elevation data were derived from the drone-based digital surface model. For the surrounding areas, LIDAR raster data from the National Land Survey of Finland was used.

The catchment areas and the flow accumulation that impact the amount of water available for re-wetting were determined using QGIS (version 3.28.6) with GRASS integration (Landa et al. 2022) and SAGA algorithm. The catchment areas were 400 hectares for Kampinneva and 440 hectares for Ohraneva.

Peat layer thickness determined by ground penetrating radar (GPR) was classified into three groups: <40 cm, 40–100 cm and >100 cm. Based on these groups different peat thicknesses were extracted from raster file into polygons representing each peat thickness level. The surface elevation and peat thickness layers were combined by using Intersect tool to show what peat thicknesses the three areas different in the adjustability of the water table had.

The third site property to consider was the mineral subsoil type and occurrence of rocks and stones. The information was obtained from the ground-penetrating radar (GPR) data. Mineral subsoil was assessed to determine agricultural suitability in areas where peat thickness was less than 40 cm.

Based on the combination of drainage and rewetting potential, peat thickness and subsoil type, the suitability of different sub-areas of the sites for various after-use types was identified.

Areas constrained to wet uses were excluded from consideration for afforestation, agriculture (including *Phalaris arundinacea*) and revegetation. Areas constrained to dry uses were excluded from consideration for peatland restoration and open-water wetland.

We considered areas with a peat layer thicker than 100 cm suboptimal for afforestation due to the need for repeated fertilization, as the tree roots cannot reach the mineral subsoil. Areas with less than 40 cm of peat and coarse-textured subsoils (sandy till or boulder-rich till) were considered unsuitable for agriculture, following the classification by Haavisto-Hyvärinen and Kutvonen (2005).

### Climate Warming Impact of the After-uses of Cutaway Peatlands

To assess the climate implications of different after-use options for cutaway peatlands, we compiled published estimates of annual fluxes of CO₂, CH₄, and N₂O across the different land use types (see Supplementary Material S1). Growing‑season balances were converted to annual values by including an assumed winter CO₂ emission equivalent to 15% of growing‑season ecosystem respiration, consistent with the approach used in the IPCC Wetlands Supplement (IPCC, 2014, Appendix 3.A.1). We used studies conducted in boreal and cool temperate climates, primarily those carried out on former peat extraction sites. This was based on a systematic review (Räsänen et al. 2023), which we updated with articles published since April 2022.

We estimated the temporal development of the greenhouse gas fluxes following land-use conversion, to the extent permitted by the available data. When the distribution of values was not normal, we used the median instead of the arithmetic mean. To represent uncertainty or variability in the estimates, we used either 95% confidence intervals or the range of emissions reported across different studies, depending on the source publications; see Supplementary Material S1 for details.

Measurement‑based GHG flux data were not available for graminoid paludiculture on former peat extraction sites. The available measurements from former agricultural peatlands were compiled for context, but these data were used only for comparison and not in the scenarios.

Based on the temporal development of the greenhouse gas fluxes, we calculated the climate warming impact of these GHG components using the REFUGE4 model (Lindroos 2023). The REFUGE4 model accounts for changes in emissions over time and the atmospheric dynamics of different gases. We applied the SSP2–4.5 ‘middle‑of‑the‑road’ baseline scenario in the calculations. Finally, we express the resulting climate-warming effect as constant carbon dioxide equivalents (CO_2_-eq) for two time periods: 16 and 100 years, starting from the year 2020. This conversion enables direct comparison of climate-warming impacts across land-use options.

In agricultural land, emissions from peat decomposition cease once the remaining peat has fully decomposed. We estimated the time required for the decomposition of a 20 cm peat layer using (i) the median of literature-based values for the dry bulk density of residual peat, 0.18 g cm⁻³ (Mäkiranta et al. 2007; Shurpali et al. 2008; *n* = 4; only sites without mineral-soil admixture), (ii) the average carbon fraction of geological peatlands in Finland, 0.523 (Turunen et al. 2002) and (iii) the carbon loss as CO_2_, CH_4_ and DOC associated with each land-use option (Maanavilja 2026).

### Climate Impact of Different Land Use Scenarios at the Case Study Sites

To assess how land use preferences influence climate impact, we developed five land use scenarios.

The *climate regulation* scenario was constructed by selecting, within the constraints imposed by site properties, the land use option with the greatest cooling or least warming effect, based on our review of climate warming impacts (Section “Climate warming impact of the after-uses of cutaway peatlands”). In all areas where it is feasible, this scenario includes short-rotation afforestation with downy birch (*Betula pubescens*). In areas constrained to wet uses, it features peatland restoration and, in the wettest depressions, open-water wetlands.

The *ecological restoration* scenario includes peatland restoration in all areas where it is feasible, open-water wetlands in the wettest depressions and natural revegetation in the areas that are constrained to dry land use. The priority order reflects the EU restoration target of preserving carbon-rich ecosystems (European Commission 2021).

The *food production* scenario prioritizes the cultivation of forage grass in all areas where it is feasible. Conventional afforestation is prioritized second, where feasible, and open-water wetlands are featured in the wettest depressions. This priority order is in line with the views of landowners in agriculture-intensive regions of Finland (Laasasenaho et al. 2023).

The *timber production* scenario prioritizes conventional afforestation in all areas where it is feasible. Cultivation of forage grass is prioritized second, where feasible, and open-water wetlands are featured in the wettest depressions. This order reflects landowner preferences in Finland (Laasasenaho et al. 2023).

The *peat-replacing biomass* *production* scenario prioritizes the cultivation of *Phalaris arundinacea* where feasible, and open-water wetlands are featured in the wettest depressions.

Climate impact of each scenario for both study sites was calculated by multiplying the area designated for each after-use type by the after-use type-specific estimate of global warming potential that was calculated for 16-year and 100-year time frames in the Section “Climate warming impact of the after-uses of cutaway peatlands”. For agricultural after-uses (grass and *Phalaris arundinacea)*, the duration of emissions was determined based on the depletion of the peat layer, as described in Section “Climate warming impact of the after-uses of cutaway peatlands”, using peat depth data derived from the GPR measurements.

## Results

### Case Study Site Properties

**Fig. 1:**
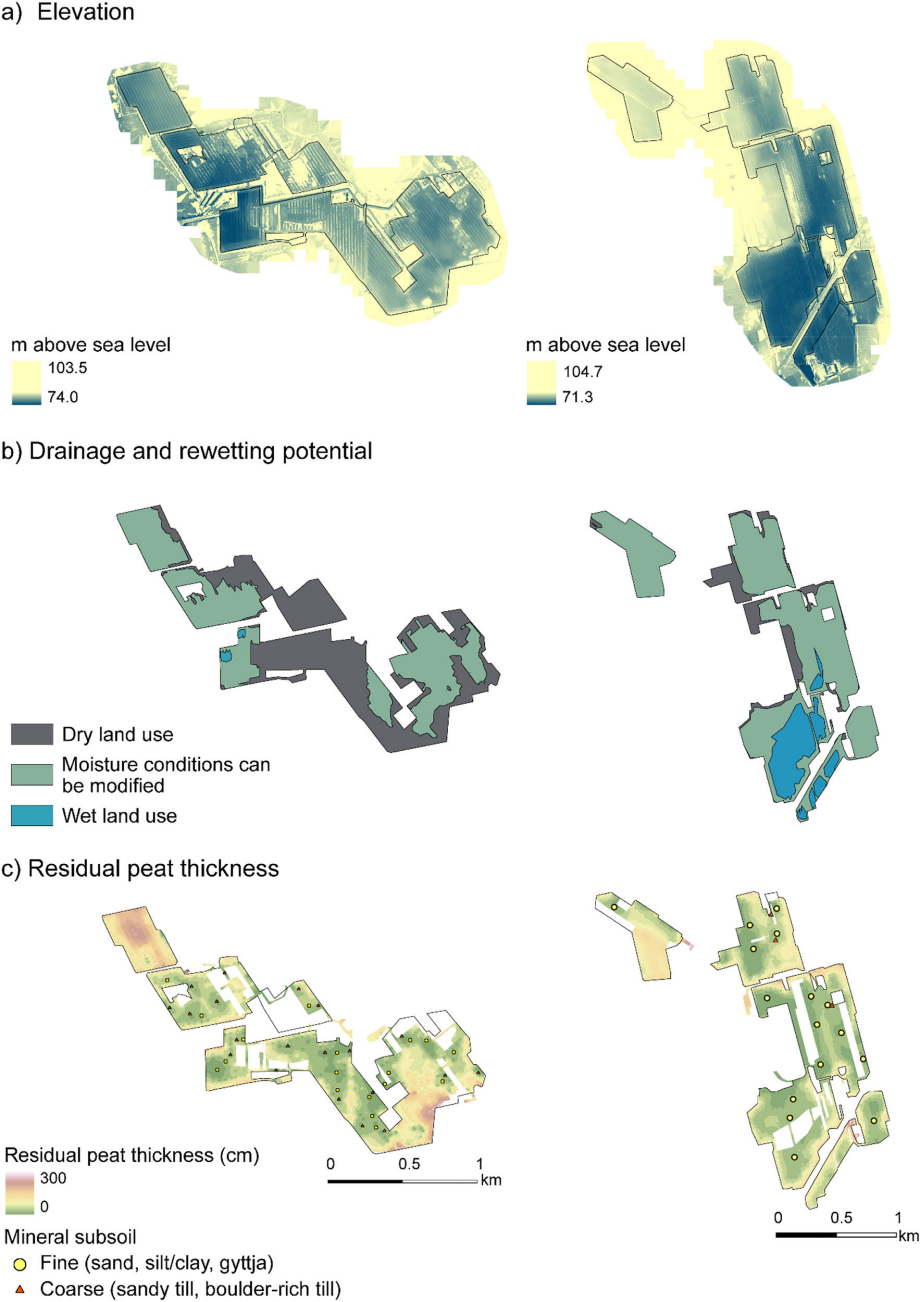
**a** Digital surface models (DSMs) of the sites Kampinneva (left) and Ohraneva (right), showing the variation in relative elevation. **b** Areas classified by their potential for dry land use and rewetting: grey areas are constrained to dry land uses, as raising the water level in these parts would affect surrounding land use, green areas can be either re-wetted or kept dry, and blue areas are difficult to keep dry due to their elevation relative to the main drainage ditch. Maximum surface levels used for areas that are difficult to keep dry were 77.8 m a.s.l. in Kampinneva (left) and 74.5 m a.s.l. in Ohraneva (right). **c** Peat thickness variation at the sites

The case study sites Kampinneva and Ohraneva exhibited substantial variation in topography (Fig. 1), which influenced the potential to manage moisture conditions. While in most of the area it was possible to establish either rewetted or dry conditions through ditch and dam design, both sites also included areas that were constrained to dry or wet uses (Fig. 1).

The residual peat layer thickness at the two sites ranged from 0 to 330 cm but was mostly less than 100 cm (Fig. 1). Both sites included areas where peat thickness was less than 40 cm, which required further investigation if agriculture is considered, as well as areas where peat thickness exceeded 100 cm, which we considered unsuitable for afforestation (Fig. 1). The subsoil consisted of sand (22% at Kampinneva, 41% at Ohraneva), sandy till (56% and 6%), silt/clay (15% and 45%), gyttja (4% and 4%) and bedrock (1% and 2%). Additionally, less than 1% comprised fine-grained till, boulder-rich till and silt. In areas with less than 40 cm of peat, several locations also contained stones.

**Fig. 2:**
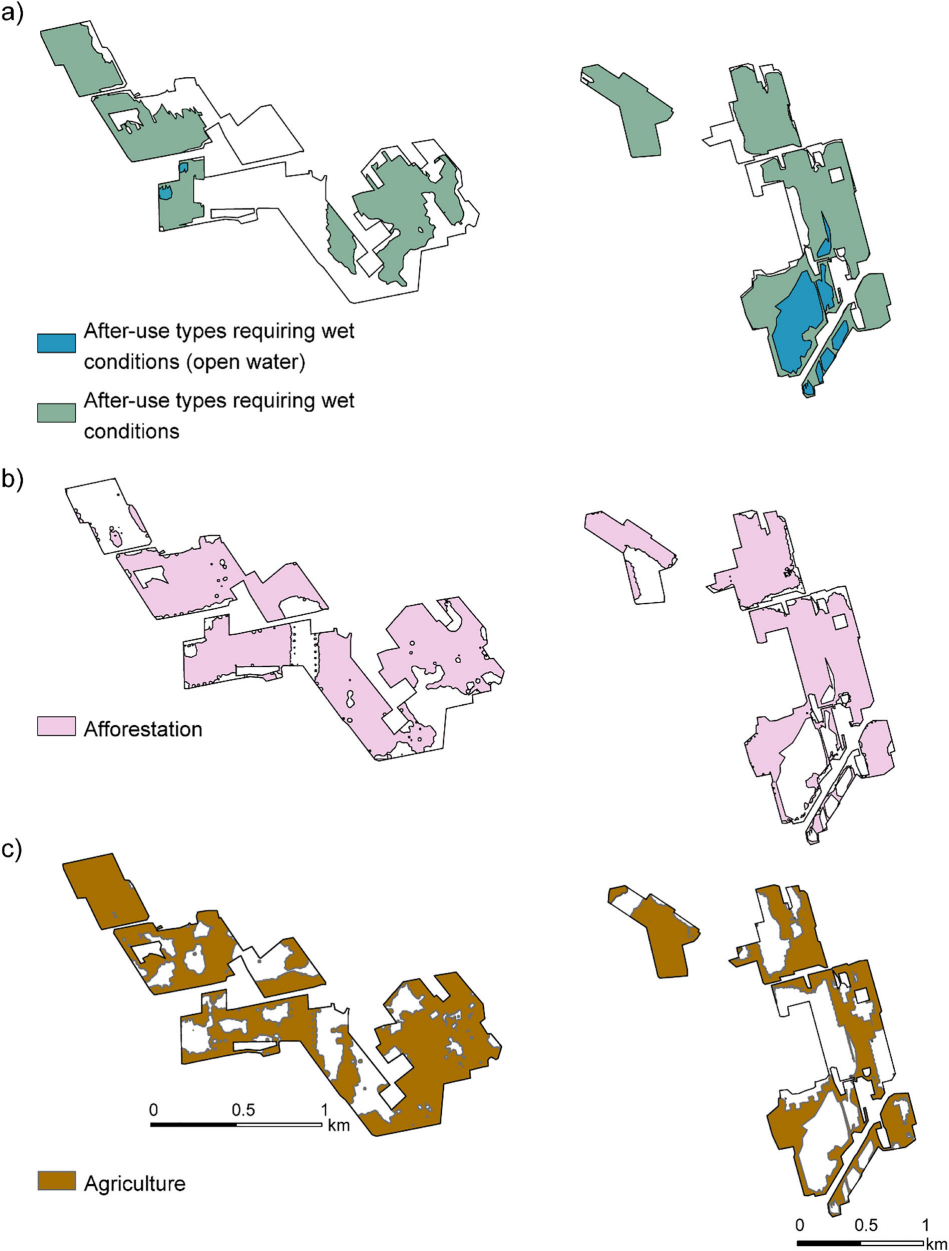
Land area suitable for after-use types requiring **a** wet conditions (restoration, paludiculture), **b** afforestation, and **c** agriculture (forage grass, *Phalaris arundinacea*) at Kampinneva (left) and Ohraneva (right)

Apart from small areas that were hard to keep dry and suitable only for wetland establishment, several after-use types were considered suitable for most of the area (Fig. 2). Areas were excluded from afforestation due to a peat layer thicker than 100 cm, and from agriculture due to the presence of thin peat combined with stones (Figs. 1 and 2).

### Carbon Balance and Climate Impact of Land-use Types

**Fig. 3:**
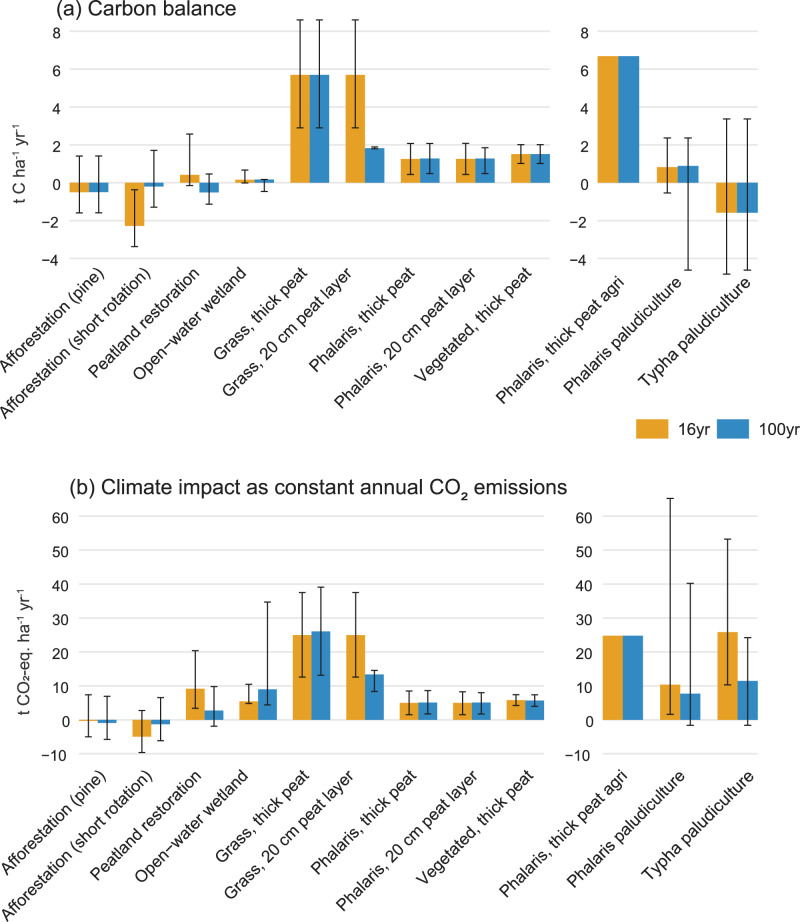
Average (**a**) annual carbon balance and (**b**) climate impact for various after-use types over 16- and 100-year time frames. Climate impact is calculated using the REFUGE4 model (Lindroos 2023) and expressed as constant annual CO_2_ emissions over 16- and 100-year time frames. Negative values indicate a CO₂ sink from the atmosphere, whereas positive values indicate a CO₂ source. The left panel shows the values used in our scenarios, while the right panel presents values from former agricultural peatlands for comparison. Error bars represent the range of values reported across studies, except for peatland restoration and grass cultivation, where they correspond to the 95% confidence intervals from Nugent et al. (2019) and IPCC (2014), respectively. For afforestation, the range of measured soil emission values is taken from the synthesis by Jauhiainen et al. (2023). For open-water wetlands, the error bars represent the range of littoral-zone emissions. Detailed GHG values and their sources are provided in the Supplementary Material S1.

Average annual carbon balances over 16- and 100-year time frames ranged from negative (carbon sink) to strongly positive values (carbon source) (Fig. 3a). A carbon sink function or near-zero balance was estimated for afforestation (pine −0.5 t C ha^−1^ yr^−1^, range −1.6 to 1.4; short-rotation birch −0.2 t C ha^−1^ yr^−1^, range −1.3 to 1.7) and for high-water-level after-use types, including peatland restoration and wetland creation (peatland restoration −0.5 t C ha^−1^ yr^−1^, range −1.1 to 0.5; wetland creation 0.2 t C ha^−1^ yr^−1^, range −0.5 to 0.2). Forage grass cultivation resulted in the highest carbon emissions (5.7 t C ha^−1^ yr^−1^, range 2.9–8.6). *Phalaris arundinacea* cultivation under drained conditions was a small carbon source at former peat extraction sites (1.3 t C ha^−1^ yr^−1^, range 0.5–2.1), as was the vegetated surface (1.5 t C ha^−1^ yr^−1^, range 1.0–2.0) (Fig. 3a).

The climate impacts of after-use types other than afforestation, were positive (i.e. climate warming), but the magnitude varied between land uses and time frames (Fig. 3b). Over the 100-year time frame, afforestation had a near-zero climate impact (−1 t CO₂-eq ha⁻¹ yr⁻¹ for both pine and short-rotation birch, range −6 to 7). Peatland restoration showed a low warming impact, with uncertainty spanning from weak cooling to moderate warming (3 t CO_2_-eq. ha^−1^ yr^−1^, range −2 to 10). Open‑water wetlands showed a moderate warming impact (9 t CO₂‑eq ha⁻¹ yr⁻¹), but with a wide range (4–35) reflecting highly variable CH₄ emissions across sites. Conventional forage grass cultivation showed the highest climate warming impact (26 t CO_2_-eq. ha^−1^ yr^−1^, range 13–39). *Phalaris arundinacea* cultivation under drained conditions and the vegetated surface showed low warming impacts (*Phalaris* 5 t CO_2_-eq. ha^−1^ yr^−1^, range 2–9; vegetated 6 t CO_2_-eq. ha^−1^ yr^−1^, range 4–7). The longer time frame (100 years) reduces the climate-warming impact (i) for peatland restoration, due to a developing CO₂ sink and the short atmospheric lifetime of CH₄, and (ii) for grass cultivation on thin peat, where the peat layer is depleted within the first decades after conversion (Fig. 3b).

Compared to former agricultural peatlands, the climate-warming impact of both dry and wet land uses at former peat extraction sites was generally low (Fig. 3b). The high warming impact of paludiculture at former agricultural peatlands over the 16-year time frame was primarily driven by CH₄ emissions, although the values reported across studies varied widely. Over the 100-year time frame, paludiculture options showed a lower warming impact than dry cultivation on former agricultural peatlands, bringing their climate warming effect to the same level as the land uses at former peat extraction sites. As a whole, most after-use types in our scenario analysis show relatively low climate-warming impacts in a European context, where agriculture is the dominant use of peatlands (Fig. 3b).

### Climate Impact of the Land Use Scenarios

**Fig. 4:**
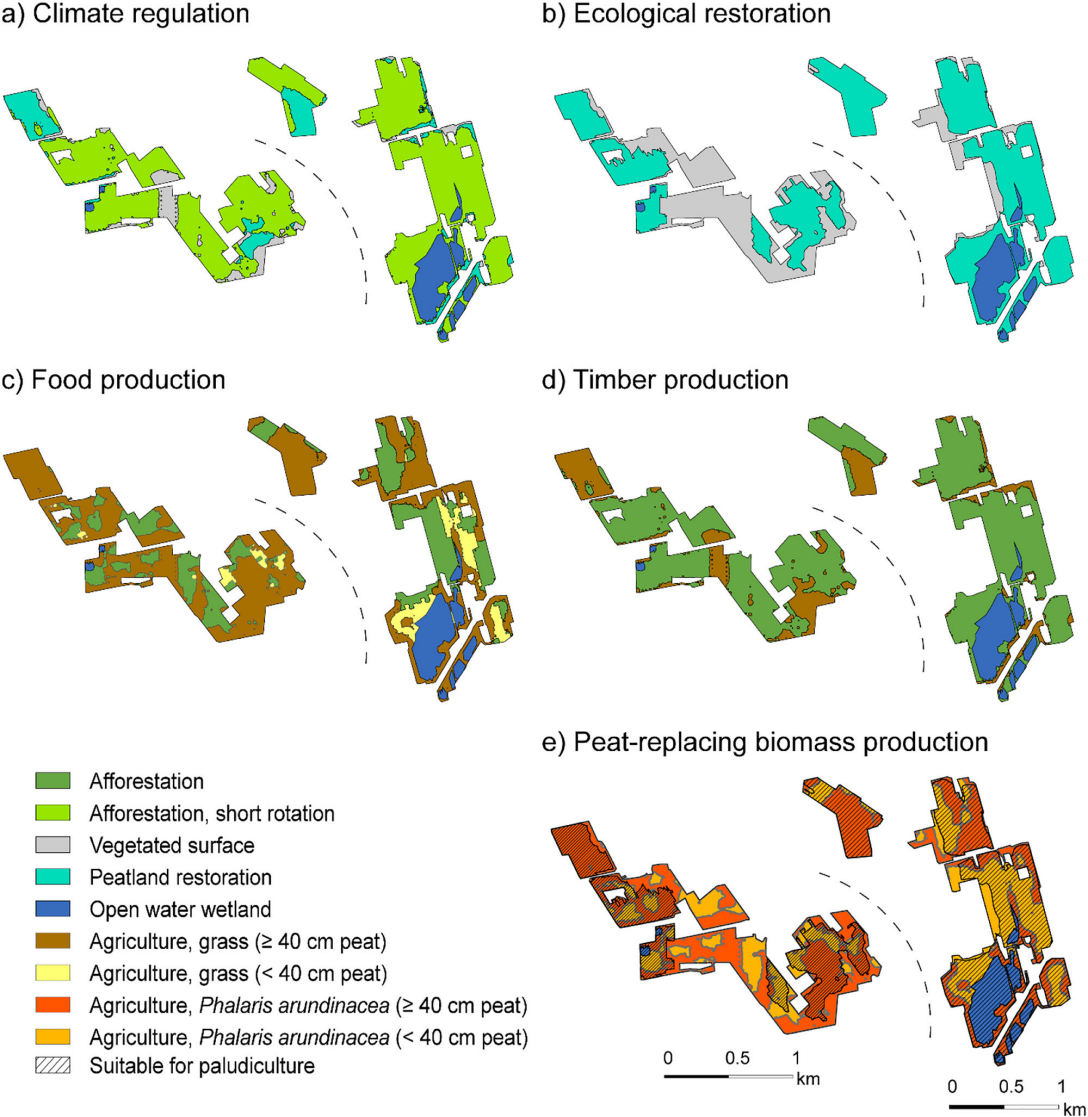
Land use scenarios aligned with different objectives for the case study sites Kampinneva and Ohraneva: **a** climate regulation, **b** ecological restoration, **c** food production, **d** timber production, **e** peat-replacing biomass production. For each scenario, land uses were allocated to areas where they are biophysically feasible, based on spatial variation in site properties. Areas suitable for paludiculture are indicated in (**e**)

The land use scenarios (Fig. 4) impacted the site-level climate outcome substantially. Over the 100-year time frame, the climate impact ranged from 30 to 2184 t CO_2_eq yr^−1^ in Kampinneva (Table 1) and from 151 to 2446 t CO_2_ eq yr^−1^ in Ohranneva (Table 1). Because the climate-regulation scenario was defined by selecting the after-use types with the lowest warming impact, it produced the most favourable climate outcome over both 16- and 100-year time frames at both sites. Over 100 years, the impacts were 0.2 and 0.9 t CO_2_eq yr^−1^ ha^−1^ for Kampinneva and Ohraneva, respectively (Table 1).

**Table 1:** Climate impact of the scenarios in the case study sites Kampinneva and Ohraneva as constant CO_2_ equivalents (t CO_2_-eq. yr^−1^) calculated for 16-year and 100-year time frames

Kampinneva		afforestation, pine	afforestation, short rotation	vegetated surface	peatland restoration	open-water wetland	agriculture, grass	agriculture, *Phalaris*	Total, t CO_2_-eq. yr^−1^	min	max	per ha
Climate regulation	area, ha		89	17	17	1						
	16 yr		−442	96	152	4			−189	−731	714	−1.5
	100 yr		−116	94	46	6			30	−513	895	0.2
Ecological restoration	area, ha			64	59	1						
	16 yr			371	537	4			912	471	1675	7.4
	100 yr			364	161	6			530	148	1071	4.3
Food production	area, ha	33				1	90					
	16 yr	−11				4	2233		2226	966	3600	18.1
	100 yr	−30				6	2208		2184	976	3039	17.7
Timber production	area, ha	89				1	33					
	16/100 yr	−29				4	829		803	−26	1910	6.5
	100/yr	−82				5	864		787	−77	1941	6.4
Peat-replacing biomass production	area, ha					1		122				
	16 yr					4		595	599	224	961	4.9
	100 yr					6		583	589	223	950	4.8

Ohraneva		afforestation, pine	afforestation, short rotation	vegetated surface	peatland restoration	open-water wetland	agriculture, grass	agriculture, *Phalaris*	Total, t CO_2_-eq. yr^−1^	min	max	per ha
Climate regulation	area, ha		122	4	18	27						
	16 yr		−602	21	168	145			−269	−968	1011	−1.6
	100 yr		−158	20	50	239			151	−653	1925	0.9
Ecological restoration	area, ha			20	123	27						
	16 yr			117	1131	145			1393	632	2942	8.2
	100 yr			114	338	239			691	−35	2281	4.1
Food production	area, ha	48				27	95					
	16 yr	−16				145	2369		2499	1082	4190	14.7
	100 yr	−44				239	2251		2446	1061	4281	14.4
Timber production	area, ha	122				27	22					
	16/100 yr	−39				145	545		650	−207	1992	3.8
	100/yr	−111				239	568		696	−299	2619	4.1
Peat-replacing biomass production	area, ha					27		144				
	16 yr					145		697	843	387	1396	5.0
	100 yr					239		673	912	375	1972	5.4

The scenarios with the next lowest warming impact were ecological restoration (4.3 and 4.1 t CO₂‑eq yr⁻¹ ha⁻¹), timber production (6.4 and 4.1), and peat‑replacing biomass production (4.8 and 5.0) (Table 1). The scenario that prioritized food production produced the highest warming impact: 17.7 and 14.4 1 t CO₂‑eq yr⁻¹ ha⁻¹ for Kampinneva and Ohranneva, respectively, over 100 years.

## Discussion

### A Variety of Climate-wise Choices Available

Our study presents a variety of land use options for former peat extraction sites with relatively low climate warming impact. The emissions of most studied land uses are lower than those measured for at peat extraction sites immediately after energy peat harvesting, for which estimates vary from 6.2 t CO_2_ ha^−1^yr^−1^ in temperate maritime Ireland (Wilson et al. 2015), to 9.5 t CO_2_ ha^−1^yr^−1^ in boreal Finland (Alm et al. 2007), and up to 10.3 t CO_2_ ha^−1^yr^−1^ according to the IPCC Tier 1 emission factor, which also includes sites extracted for horticulture (IPCC 2014). In addition, for the same crops—such as *Phalaris arundinacea* or timber-producing tree stands—the greenhouse gas emissions are generally lower on former peat extraction sites than on peatlands with a drainage legacy of agriculture or forestry (Jauhiainen et al. 2023; Kandel et al. 2013). This is likely due to the properties of peat following energy peat extraction: the typically thin residual peat layer is highly decomposed and therefore resistant to further decay (Glatzel et al. 2004).

The different scenarios studied here address the key objectives associated with the after-use of peat extraction sites—namely, mitigating climate change, halting biodiversity loss, and generating income for landowners and the local economy (European Parliament and Council of the European Union 2021). Our two case examples suggest that a landowner who adopts a combination of afforestation, peatland restoration, open-water wetlands and vegetated areas can achieve a near-climate-neutral outcome over a 100-year time frame. Prioritizing ecological restoration, timber production, or biomass production for growing media or animal bedding results in a mild climate-warming effect. Prioritizing food production causes the greatest climate warming among the studied scenarios.

The *climate regulation scenario* features a substantial share of short-rotation forestry with a favourable climate change mitigation impact. However, these kinds of monoculture stands with even-aged trees support low biodiversity compared to natural forests (Griffiths et al. 2018), and do not safeguard the long-term peat storage or the geodiversity (Gray 2013) of the landscape. As part of the landscape, though, short-rotation downy birch stands may provide nesting sites for passerine birds and serve as winter food sources for woodland grouse (Kiljunen et al. 2024). For landowners, short-rotation forestry has the potential to provide a reliable and continuous source of income (Jylhä et al. 2015). Both conventional and short-rotation forestry require effort and inputs from the landowner to be successful, most importantly wood ash fertilization, even in favourable areas with thin peat where the tree roots are able to reach the mineral subsoil eventually (Hytönen and Aro 2012; Aro et al. 2020).

The *ecological restoration scenario* preserves and restores the geodiversity of the landscape through peatland restoration and enhances landscape-level biodiversity by supporting peatland and wetland species that are increasingly endangered due to widespread drainage (Minayeva et al. 2016, Hyvärinen et al. 2019). In areas where rewetting is not feasible, the establishment of vegetation cover provides habitats initially supporting early successional species, with the subsequent successional trajectory remaining uncertain (Huotari et al. 2007). However, the climate impact of this scenario is less favourable than that of afforestation-based climate scenario over a policy-relevant timescale. Although peatland restoration safeguards the carbon storage, initial emissions from decomposing straw used to protect the transplanted moss and methane emissions reduce the short-term climate benefit (Nugent et al. 2019). Over a 100-year timescale, the net climate impact becomes nearly neutral, approaching that of afforestation. Furthermore, the extensive vegetated surface area on land that is too dry for peatland restoration contributes to a lower overall climate benefit compared to afforestation.

The *timber production* and *the food production scenarios* align with the conventional after-use practices in Finland, providing livelihood and income for the landowners. The inclusion of forage grass cultivation worsens their climate impact, making the food production scenario the least favourable in terms of climate outcomes. From a biodiversity perspective, neither scenario is ideal: while both increase biodiversity compared to an active peat extraction site, they provide limited benefits at broader landscape scales, as they primarily support habitat conditions typical of managed forests and agricultural land—communities that are already widespread in Finland and Europe (Rana et al. 2024).

The *peat-replacing biomass scenario* includes cultivation of *Phalaris arundinacea*. Compared to conventional agriculture, its climate-warming impact is substantially lower. *Phalaris arundinacea* may provide habitats for birds, although its dense stands are not favourable for species that prefer open habitats (Vepsäläinen 2010). For landowners, in regions where demand for *Phalaris arundinacea* biomass for fibre applications such as bedding or growing media exists, its cultivation may provide a viable income opportunity (Jylhä et al. 2026).

All scenarios studied here include the same areal coverage of open-water wetlands located in the lowest depressions of the sites—areas that would require continuous water pumping to remain dry. Creating open-water wetlands in low-lying areas is likely to provide landscape-level benefits for biodiversity, water quality and water retention (Ghermandi et al. 2010; Kačergytė et al. 2021). Although open-water wetland creation results in higher emissions than peatland restoration, it remains a moderately climate-friendly option for areas where water naturally accumulates. These wetlands are often maintained as open water to support avian populations (Alhainen et al. 2015), which is the assumption used in our scenarios. However, if allowed to follow natural succession—and depending on local hydrological conditions—open water wetlands may eventually succeed to *Sphagnum*-dominated mires (Korhola 1992; Leppälä et al. 2008), thereby improving their climate impact.

The wide range of climate-wise after-use options allow landowners and planners to select or promote land uses that best suit the characteristics of each site. However, in practice, these choices are often constrained by national legislation, policy conventions, and cultural norms. While cultural and regional diversity in land use practices should be respected, there is also significant potential for countries to learn from one another regarding after-use strategies, legislation, and subsidy schemes. For example, in Finland, *Phalaris arundinacea* cultivation with winter harvest is not currently eligible for agricultural subsidies as an after-use on newly cleared lands, despite its potential as a low-emission biomass crop (Finnish Food Authority 2025). Similarly, in Estonia, any type of fertilization, also ash that is a prerequisite for successful afforestation and efficient carbon uptake of the tree stands on former peat extraction sites, is not supported under existing regulations (Estonian Forest Act §27(3) 2013).

### Embracing Spatial Diversity in Rewetting and Land-use Planning of Former Peat Extraction Sites

The case study sites presented here illustrate that the feasibility of rewetting and the thickness of residual peat can vary considerably within a single peat extraction site. Peatlands extracted for energy tend to be less flat and, therefore, less suitable for uniform rewetting than those extracted for horticultural peat (Money 2004), such as those found in Canada, where rewetting and moss transfer is the default after-use method (González and Rochefort 2014). Recognizing these spatial variations is important when planning climate-wise and cost-effective land use (Aro et al. 2023). While technically (open water) wetlands can be constructed anywhere through earthmoving works with excavation, this approach may be costly and results in CO₂ emissions from the disturbed peat. Damming and water flow design offer a more affordable and climate-friendly alternative. These methods enable the creation of a multi-habitat landscape, tailored to the specific conditions of each sub-area. Peat extraction sites often span extensive areas: for example, in North Ostrobothnia, Finland, the average size was found to be 195 hectares (Karjalainen et al. 2024), meaning that the individual sub-areas are typically large enough to support targeted land-use practices.

Embracing spatial diversity could benefit the provision of ecosystem services, biodiversity conservation, and the current transition in policy implementation. From an ecosystem services perspective, different after-uses, if well planned, function synergistically. An open-water wetland supports a restored *Sphagnum* mire by providing a stable water source required by establishing *Sphagnum* (Oestmann et al. 2022), and the resulting wetland-mire complex may help reduce organic carbon loading from the afforested area into nearby watercourses (Borgström et al. 2024). Additionally, for avian populations, a network of smaller wetlands may be more beneficial than a single large one by enhancing breeding success (Kačergytė et al. 2021).

For landowners, rewetting may be more attractive if the rewetted area is not too extensive and if they can still pursue their original land-use plans, such as afforestation (Laasasenaho et al. 2023), in the part of area most suitable for that land use. Large areas of peat extraction sites in Finland, Estonia, Latvia, Ireland, Germany, Sweden and Canada are currently transitioning or have already transitioned to after-use (Räsänen et al. 2023).

### Knowledge Gaps in Climate Impacts of the After-use Options

The extent of scientific data available on the environmental impacts of the different after-use types varies, and substantial knowledge gaps exist (Räsänen et al. 2023). In this study, we focused on climate impacts and compiled the available published data on greenhouse gas (CO_2_, CH_4_, N_2_O) exchange associated with various after-use types. It is evident that the available data are derived from a limited number of sites—or from sites that differ fundamentally from former peat extraction sites—which limits their representativeness and introduces uncertainty in broader climate impact assessments (see Supplementary Material S1).

For afforested areas, soil GHG flux estimates are based on measurements conducted at six sites over three growing seasons (Mäkiranta et al. 2007), while estimates of carbon sequestration by the tree stands rely on data from a few pine stands under normal rotation (Aro and Kaunisto 2003; Aro et al. 2016) and a few downy birch stands of varying ages under short rotation forestry (Hytönen and Aro 2012; Hytönen et al. 2018). Measurements from a larger number of sites would be needed to ensure representativeness and to capture variability across different site conditions.

The peatland restoration estimates presented in this study are based on Canadian time series data (Nugent et al. 2019), supported by similar findings from Estonia and Finland (Järveoja et al. 2016a; Kivimäki et al. 2008; Purre et al. 2019). The greatest uncertainty lies in the first-year emissions when straw is applied, as its rapid decomposition leads to high CO_2_ emissions (Nugent et al. 2019). These emissions are sensitive to the amount of straw used and may not be relevant in all climate conditions, as straw mulch may not be required in cooler, more humid climates (Corson and Campbell 2013).

Open-water wetlands, particularly their littoral zones, are among the most studied of the after-use options. Methane emission estimated in our study is consistent with other estimates for similar habitats: from ten years post-restoration onwards, our estimate of the methane emissions from the open-water wetland matches the 2019 IPCC Refinement emission factor for freshwater and brackish ponds, as well as the IPCC Wetland Supplement emission factor for rich rewetted peatlands, at 0.14 t CH₄-C ha⁻¹ yr⁻¹ (IPCC 2019; IPCC 2014).

Grass cultivation is a common after-use in agricultural regions such as western Finland (Laasasenaho et al. 2023), yet, to our knowledge, no studies have specifically examined greenhouse gas emissions from forage grass or cereal cultivation on former peat extraction sites. In the absence of such data, we applied IPCC emission factors for organic soils in boreal and temperate/boreal zones (IPCC 2014). However, recent evidence suggest that initial soil conditions continue to influence greenhouse gas emissions from agricultural soils (Holzknecht et al. 2025). Empirical measurements from conventional agriculture on former peat extraction sites would be needed to establish a reliable baseline in regions where agriculture is a commonly pursued after-use.

The GHG exchange of *Phalaris arundinacea* has been studied over multiple years in the former boreal peat extraction site of Linnansuo (Hyvönen 2015), and measurements have also been conducted in Estonia (Järveoja et al. 2016b; Mander et al. 2012). However, the GHG exchange during the renewal year remains a knowledge gap.

Paludiculture of graminoid species for biomass production has been shown to reduce greenhouse gas emissions compared to conventional dryland cultivation on peatlands that have been drained for agricultural use – common in northern Germany, Denmark and the Netherlands (Bianchi et al. 2021; Tanneberger et al. 2022; van den Berg et al. 2024; Nielsen et al. 2024a), although flooding poses a risk of high methane emissions (Kandel et al. 2020). The greenhouse gas balance of *Phalaris arundinacea* paludiculture is shown to be highly dependent on the level and stability of water table depth (Kandel et al. 2019, 2020; Karki et al. 2019, Nielsen et al. 2024a). When the water table is precisely regulated, certain productive paludiculture systems have even been observed to produce a climate cooling effect (Bockermann et al. 2025).

However, greenhouse gas fluxes from graminoid paludiculture on former peat extraction sites remain a knowledge gap. Because their pH and peat properties differ from those of agricultural peatlands (Apori et al. 2023), the emission dynamics are potentially different. Supporting this, Nielsen et al. (2024b) found that both the biomass production and the climate impact of *Phalaris arundinacea* paludiculture were affected by peat pH and nutrient availability, with greater GHG reductions observed on high-pH, nutrient-rich sites.

The difference has several possible pathways. First, low nutrient availability limits crop carbon sequestration (Nielsen et al. 2024b). Second, raising the water table has a smaller mitigating effect on peat decomposition when nutrient availability is low, since decomposition is already slow in the drained state; this pattern is supported by cross‑site analyses in forestry‑drained peatlands (Ojanen and Minkkinen 2019). Consequently, at former peat extraction sites, it seems unlikely that graminoid paludiculture would provide a substantial additional climate benefit for *Phalaris arundinacea* cultivation within a policy-relevant time frame. In fact, in our results, the dry cultivation at former peat extraction sites has a lower or comparable climate warming impact than paludiculture at former agricultural peatlands.

In contrast, *Sphagnum* farming paludiculture could be a viable, climate-wise option for former peat extraction sites. It likely results in a similar climate impact than mire restoration (Beyer and Höper 2015). While its GHG exchange has been studied to some extent (Beyer and Höper 2015; Oestmann et al. 2022), time series covering the full harvest cycle are still lacking. While we did not include *Sphagnum* farming in our scenarios, it can be considered analogous to peatland restoration with *Sphagnum* transfer (Nugent et al. 2019), with the key difference that eventual biomass harvest must be accounted for. The typically low nutrient availability and pH of residual peat make these sites more suitable for *Sphagnum* establishment than agricultural peatlands, although water table regulation on the dense, highly decomposed peat remains a challenge (Oestmann et al. 2022; Wichmann et al. 2017).

In some countries, like Finland, establishing a green vegetation surface is the minimum management option and required by environmental permits (Aro et al. 2023). The vegetation establishment is low-cost and commonly done with ash fertilization. It helps prevent wind erosion and nutrient leaching while initiating a vegetation CO₂ sink (Ots et al. 2024). Despite its widespread use, no comprehensive GHG measurements are currently available for this practice. In the absence of better data, in here we used values from unfertilized *Phalaris arundinacea* (Järveoja et al. 2016b) and abandoned sites (Tuittila et al. 1999, 2000). Since ash fertilization enhances vegetation growth, the actual climate impact may be more favourable than our estimate suggests. Measured data would be highly valuable, as this practice could also serve as a baseline for comparing the climate impacts of other after-use options.

## Conclusions

While it is clear that the climate impacts of most post-extraction land use types require further research, our review suggests multiple viable pathways toward sustainable climate outcomes. The physical heterogeneity within peat extraction sites calls for spatially tailored land-use strategies, which are likely to deliver greater ecosystem service benefits and be more cost-effective than uniform approaches. Our results suggest that both afforestation and rewetting can mitigate the climate‑warming impact of former peat extraction sites over a 100‑year time frame, although more measurements from afforested sites under varying soil and hydrological conditions are needed to strengthen this conclusion.

## Supplementary information

Suppl_S1_PeatCutawayProperties

## Data Availability

Data are provided in a supplementary information file. Supplementary Material S1 Excel file containing the compiled greenhouse gas flux estimates and the calculated climate warming impacts.

## Code Availability

The peat‑depletion‑based calculation method is available open access on Zenodo (Maanavilja 2026).
